# Probiotics improve the neurometabolic profile of rats with chronic cholestatic liver disease

**DOI:** 10.1038/s41598-021-81871-8

**Published:** 2021-01-26

**Authors:** Veronika Rackayová, Emmanuelle Flatt, Olivier Braissant, Jocelyn Grosse, Daniela Capobianco, Paola Mastromarino, Matthew McMillin, Sharon DeMorrow, Valérie A. McLin, Cristina Cudalbu

**Affiliations:** 1 Laboratory for Functional and Metabolic Imaging, Ecole Polytechnique Fédérale de Lausanne (EPFL), Lausanne, Switzerland; 2 Service of Clinical Chemistry, University of Lausanne and University Hospital of Lausanne, Lausanne, Switzerland; 3 Laboratory of Behavioral Genetics, Ecole Polytechnique Fédérale de Lausanne (EPFL), Lausanne, Switzerland; 4 Department of Public Health and Infectious Diseases, Section of Microbiology, Sapienza University of Rome, Rome, Italy; 5 Department of Internal Medicine, Dell Medical School, The University of Texas at Austin, Austin, TX USA; 6 Central Texas Veterans Health Care System, Temple, TX USA; 7 Division of Pharmacology and Toxicology, College of Pharmacy, The University of Texas at Austin, Austin, TX USA; 8 Swiss Pediatric Liver Center, Department of Pediatrics, Gynecology and Obstetrics, University of Geneva and University Hospitals Geneva, Geneva, Switzerland; 9 Center for Biomedical Imaging, Ecole Polytechnique Fédérale de Lausanne (EPFL), Lausanne, Vaud Switzerland

**Keywords:** NMR spectroscopy, Liver diseases, Hepatology, Experimental models of disease, Preclinical research, Neurological disorders

## Abstract

Chronic liver disease leads to neuropsychiatric complications called hepatic encephalopathy (HE). Current treatments have some limitations in their efficacy and tolerability, emphasizing the need for alternative therapies. Modulation of gut bacterial flora using probiotics is emerging as a therapeutic alternative. However, knowledge about how probiotics influence brain metabolite changes during HE is missing. In the present study, we combined the advantages of ultra-high field in vivo ^1^H MRS with behavioural tests to analyse whether a long-term treatment with a multistrain probiotic mixture (VIVOMIXX) in a rat model of type C HE had a positive effect on behaviour and neurometabolic changes. We showed that the prophylactic administration of this probiotic formulation led to an increase in gut *Bifidobacteria* and attenuated changes in locomotor activity and neurometabolic profile in a rat model of type C HE. Both the performance in behavioural tests and the neurometabolic profile of BDL + probiotic rats were improved compared to the BDL group at week 8 post-BDL. They displayed a significantly lesser increase in brain Gln, a milder decrease in brain mIns and a smaller decrease in neurotransmitter Glu than untreated animals. The clinical implications of these findings are potentially far-reaching given that probiotics are generally safe and well-tolerated by patients.

## Introduction

Chronic liver disease (CLD) is often accompanied by type C hepatic encephalopathy (HE) which becomes apparent as liver disease progresses or as patients experience precipitating factors.

Ammonium is known to play a central role in the pathogenesis of HE^1^. The main source of systemic ammonium is believed to be the gut, partially from urea breakdown by urease producing colonic bacteria and glutamine deamidation by glutaminase^2,3^. In cirrhotic patients, it has been shown that urease producing bacteria are increased^4,5^ leading to increased urea breakdown and ammonia absorption^6^.

There is increasing evidence that chronic liver disease is accompanied by qualitative and quantitative changes in the intestinal flora. Changes in gut microbiota in CLD include a decrease in autochthonous and an increase in potentially pathogenic bacteria, which become more pronounced with progression of liver disease^7–9^. Dysbiosis and bacterial overgrowth both of which are present in cirrhotic patients^9–11^ can lead to increased production of endotoxins^8,12–14^ entering the circulation^15^. Furthermore, dysbiosis and secondary intestinal inflammation^16–18^ may lead to increased gut permeability, in turn leading to bacterial translocation^19,20^. Together, this increases systemic inflammation^8,21^ and further contributes to the progression of liver disease and its complications. It is recognized that modifications in gut flora are implicated in the complications of liver cirrhosis or CLD, including spontaneous bacterial peritonitis (SBP) and other infections^22,23^, all of which are considered precipitating factors of HE.

Non-absorbable disaccharides (e.g. lactulose or lactitol) or non-absorbable antibiotics such as rifaximin are the most commonly used therapies in HE. The mechanisms of action of these treatments are not yet fully understood, but the main effect is probably linked to changes in gut bacterial metabolic functions and microbiome composition (mainly through decrease ammonia production in the gut and its absorption)^24,25^. However, these treatments have some limitations in their efficacy and tolerability, emphasizing the need for alternative therapies. Modulation of gut bacterial flora using probiotics is emerging as a therapeutic alternative. Not only may they present additional benefits, but they are also generally well tolerated clinically^26,27^.

If the right strains of probiotic bacteria are used, a decrease of pathogenic bacteria will lead to decreased production of gut-derived bacterial toxins^28^ and ammonium^29–31^ and also help restore intestinal barrier integrity^32^. A probiotic formulation consisting of a mixture of eight strains (VIVOMIXX in EU, VISBIOME in USA) has been associated with significant improvement in minimal HE (mHE) symptoms in humans^33^, decreasing hospitalization rates and preventing HE episodes in patients with cirrhosis^34^. It has also been shown recently that this probiotic formulation improved cognitive function and inflammatory response in patients with cirrhosis^35^. In general, the effect of probiotics was similar to lactulose^36–38^ but with an improved tolerability profile. It should be noted that there was a wide variability in type of strain, daily dose and length of treatment in most of previously published studies, limiting the interpretation of treatment efficacy.

Type C HE is associated with neurometabolic changes in patients. These are characterized by an increase in brain glutamine (measured as the sum of glutamine and glutamate at low magnetic fields) due to ammonium detoxification, and consequent decrease of other brain metabolites, such as myo-inositol or choline-containing compounds^39,40^. It was also shown that these changes may underlie neuropsychiatric impairment^41^.

In animal models of type C HE, more detailed neurometabolic changes have been measured using longitudinal in vivo ^1^H MRS or a combination of ^1^H MRS and ^31^P MRS at ultra-high field (9.4 T), including changes in antioxidant, neurotransmitter and energy metabolites^39,42,43^. Yet, the studies evaluating the effects of probiotics are sparse in type C HE animal models. D’Mello et al. reported that probiotics (formulation sold as VSL#3 until 2016, but now exclusively available under the brands VIVOMIXX and VISBIOME) improved inflammation-associated sickness behaviour in a mouse model of liver inflammation^44^. One study reported reduced liver fibrosis and hepatic gene expression of Interleukin-6 (IL-6) in BDL rats when using the probiotic *Lactobacillus rhamnosus* GG^45^.

To date, the efficacy of probiotics on HE has mainly been assessed using neurological testing^46^. Knowledge about how probiotics influence brain metabolite changes, commonly present during HE, is missing. Therefore, our study aimed to use the advantages of ultra-high field in vivo ^1^H MRS combined with behavioural tests to analyse whether a long-term treatment with a multistrain probiotic mixture (VIVOMIXX) in a rat model of type C HE attenuated the behavioural and neurometabolic changes typically observed in this model. We focused specifically on brain metabolites (i.e. glutamine and glutamate separately, taurine, creatine, γ-aminobutyric acid, phosphocreatine, ascorbate or glutathione) involved in osmoregulation, neurotransmission, energy or antioxidant metabolism.

## Methods

Adult male Wistar rats (Charles River laboratories, L’Arbresle, France, 160–195 g) underwent bile duct ligation (BDL) or sham operation to reproduce an accepted model of type C HE^47^. Animals were kept in the animal facility with 12 h/12 h light/dark cycle. Standard rat chow and water was available ad libitum for the duration of study. All animal experiments were conducted according to federal and local ethical guidelines, and the protocols were approved by the local Committee on Animal Experimentation for the Canton de Vaud (Switzerland).

### Study design

In the present study, thirty-eight rats were separated into 4 groups: 14 BDL rats with probiotic administration (BDL + probiotic), 14 BDL rats without treatment (BDL), 5 sham operated animals with probiotic administration (sham + probiotic) and 5 sham operated animals without treatment (sham). Of note, the “BDL” rats and the measures performed using these rats are part of a previously published study^43^.

Daily probiotic administration started 2 weeks before BDL or sham surgery and lasted until the end of the study (8 weeks post-BDL). The dose of 60 billion probiotic bacteria/kg_rat_/day was dissolved in saline solution and given by voluntary drinking from a syringe (without needle). The group without treatment received the same volume of saline solution. As a probiotic mixture we used VIVOMIXX (Mendes S.A., Lugano, Switzerland) containing 8 lyophilized, highly viable bacterial strains: 4 lactobacilli (*Lactobacillus acidophilus* DSM24735, *L. plantarum* DSM24730, *L. paracasei* DSM24733, *L. bulgaricus* DSM24734), 3 *Bifidobacteria* (*Bifidobacterium infantis* DSM24737, *B. longum* DSM24736, *B. breve* DSM24732) and *Streptococcus thermophilus* DSM24731).

The longitudinal study design with the timing of various sample collections or measurements is illustrated in Table 1 During the MR experiments and blood sampling (from the sublingual vein at the beginning of the afternoon), animals were kept under 1.5–2% isoflurane anaesthesia (in a mixture of 50% air and 50% oxygen) with respiration rate maintained at 60–70 breaths/min and body temperature at 37.5–38.5 °C.

**Table 1:** Longitudinal study design.

	Week − 2	Week 0 BDL/sham surgery	Week 2	Week 4	Week 6	Week 8
	Stool collection Start of probiotic treatment	Blood sampling Stool collection MRS scan	Blood sampling Stool collection	Blood sampling Stool collection MRS scan Open field	Blood sampling Stool collection MRS scan Open field	Blood sampling Stool collection MRS scan Open field Sacrifice and organ collection
n_BDL_ _non-treated_	B:6	MRS:14/B:6		MRS:12/B:5 O-F:7	MRS:8/B:5 O-F:13	MRS:10/B:4 O-F:10
n_BDL+probiotic_	B:10	MRS:14/B:10		MRS:14/B:10 O-F:10	MRS:14/B:10 O-F:14	MRS:7/B:6 O-F:5
n_sham_	B:0	MRS:5/B:0		MRS:0/B:0 O-F:5	MRS:0/B:3 O-F:5	MRS:5/B:0 O-F:4
n_sham+probiotic_	B:4	MRS:5/B:4		MRS:5/B:3 O-F:3	MRS:5/B:3 O-F:5	MRS:4/B:2 O-F:5

The number of rats (n) measured in every group are indicated for each week and each type of measures: ‘MRS’ stands for MRS scan, ‘B’ for the rats whose *Bifidobacteria* in the faeces were analysed, and ‘O-F’ for the rats who undergone open field test. Of note, for blood sampling we used the same number of rats as for the MRS scan and n_sham_ = 5 at each week (data not included in the table for readability).

### Gut microbiota analysis

Faeces were collected at weeks 0, 4 and 8 to measure *Bifidobacteria* concentration. Measurements were performed as described previously^48^.

### Plasma analysis

Plasma samples were analysed using REFLOTRON System for glucose, INTEGRA 400 Plus for ammonium and COBAS 8000 for total bilirubin as markers of biliary obstruction and liver function (Roche, Switzerland).

### ^1^H MRS

Measurements were conducted on a horizontal actively shielded 9.4 T system (Magnex Scientific, Oxford, UK) interfaced to a Varian Direct Drive console (Palo Alto, CA, USA) as previously described^43^. ^1^H localized spectra were acquired with the ultra-short echo-time SPECIAL^43,49^ spectroscopy sequence (TE = 2.8 ms, TR = 4 s, 160 averages) in a volume of interest (VOI = 2 × 2.8 × 2 mm^3^) placed in the dorsal hippocampus. Hippocampus, as a part of the limbic system, was chosen for ^1^H MRS measurements due to known problems with learning and memory in HE patients^50^.

Spectra were fitted and metabolite concentrations were calculated by LCModel and expressed in mmol/kg_ww_ using the unsuppressed water signal from the same VOI as an internal reference, assuming 80% water in the tissue, as previously described^43^. The Cramer–Rao lower bounds (CRLB) were used as a reliability measure for the metabolite concentration estimate. Only metabolites with CRLB lower than 30% were considered for further analysis. The ultra-short echo-time MRS allowed the detection of the following 18 metabolites, all included in basis-set: alanine (Ala), ascorbate (Asc), aspartate (Asp), glycerophosphocholine (GPC), phosphocholine (PCho), creatine (Cr), phosphocreatine (PCr), γ-aminobutyric acid (GABA), glucose (Glc), glutamine (Gln), glutamate (Glu), glutathione (GSH), myo-inositol (mIns), lactate (Lac), *N*-acetylaspartate (NAA), *N*-acetylaspartylglutamate (NAAG), phosphoethanolamine (PE) and taurine (Tau).

Changes of brain metabolites over time were expressed in absolute values (mmol/kg_ww_) and also as % changes. Our non-invasive method allows us to scan the animals before BDL and to follow the same animals longitudinally. Thus, the metabolic changes in the brain during the progression of disease could be compared to the pre-BDL scan and expressed as % changes in each animal for all the other time-points.

### Behavioural tests—open field test

Locomotor activity was assessed in the open field (OF) test as previously described^43^.

### Statistical analysis

All results are presented as mean ± SEM. One-way ANOVA (Prism 5.03, Graphpad, La Jolla CA USA) with the Bonferroni’s multi-comparisons post-test (weeks post-BDL, 6 comparisons) were used to assess significance in each brain and plasma metabolite’s, *Bifidobacteria* and behavioural measurement within a single group (*p < 0.05; **p < 0.01; ***p < 0.001). Gut microbiota analyses were performed on data in logarithmic scale. Two-way ANOVA (Prism 5.03, Graphpad, La Jolla CA USA) followed by the Bonferroni's multi comparisons post-test was used to assess significance (p < 0.05) of changes in each brain metabolite between the groups (with weeks post-BDL and groups as factors). However, given the difficulty of interpreting the p values of two-way ANOVA when interaction is significant (treated rats showing changes different from BDL rats in some measurements, as expected), one-way ANOVA was also used to evaluate differences between BDL non-treated and BDL + probiotic in a given week. P values are shown in tables and figures only.

## Results

### Gut microbiota: *Bifidobacteria* increase in BDL + probiotic rats

**Figure 1:**
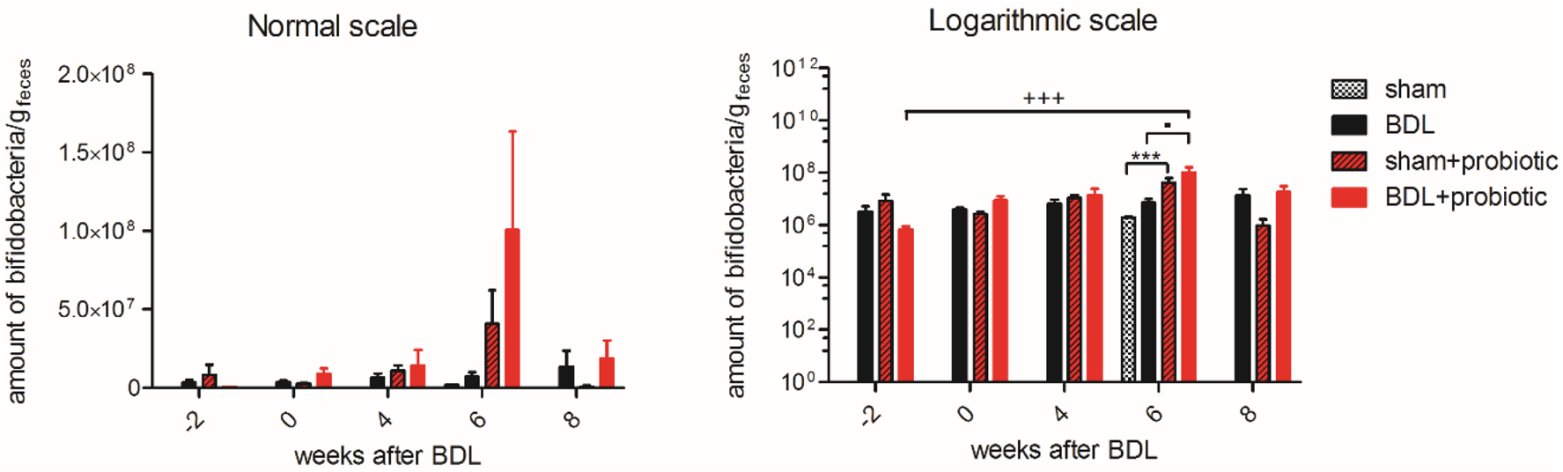
Amount of *Bifidobacteria*/g of faeces expressed in normal and logarithmic scale. One-way ANOVA was used for statistical analysis, shown on the logarithmic scale graph: *between sham and sham + probiotics at week 6, ▪between BDL and BDL + probiotic at week 6, + between BDL + probiotic between week − 2 and week 6.

Gut microbiota (Fig. 1) showed a significant increase of the amount of *Bifidobacteria* between week -2 (beginning of probiotic treatment) and week 6 in BDL + probiotic and a trend toward an increase in sham + probiotic. At week 6, both BDL + probiotic and sham + probiotic showed a significantly higher level of *Bifidobacteria* as compared to BDL or sham, respectively. No significant change was observed in BDL rats throughout the study.

### Plasma measurements and body weight: differences between shams and BDL rats

**Plasma bilirubin**, a marker of liver disease, was undetectable (< 0.5 mg/dl) in all animals before surgery and stayed undetectable in shams (with or without probiotic) throughout the study. In both BDL and BDL + probiotic groups, plasma bilirubin increased already 2 weeks post-BDL and continued to increase until week 8 without significant difference between the two groups (Table 2). **Plasma NH**_**4**_^**+**^ was in the normal range before surgery in all animals and stayed normal in shams, with no difference between shams and shams + probiotic over the course of the study (Table 2). Plasma NH_4_^+^ at week 6 and 8 was slightly lower in the BDL + probiotic group when compared to the BDL group, although this did not reach statistical significance. **Plasma glucose** was in the same range before the surgery in all animals and remained in the normal range in both in shams and shams + probiotic throughout the study. In BDL and BDL + probiotic rats plasma glucose levels decreased progressively over the course of the disease (Table 2). Even though BDL and BDL + probiotic had lower **body weight** than shams and shams + probiotic, the difference did not reach statistical significance. At week 8, mean body weights were: 299 ± 10 g in BDL group, 336 ± 16 g in BDL + probiotic group, 352 ± 17 g in sham group and 363 ± 13 g in sham + probiotic group.

**Table 2:** Evolution of plasma glucose, NH_4_^+^ and bilirubin in BDL, BDL + probiotic, sham and sham + probiotic animals during disease progression.

	BDL	BDL + probiotic	Sham	Sham + probiotic	p value
**Plasma glucose (mg/dl)**					
Week 0	189 ± 6	193 ± 6	176 ± 19	192 ± 3	ns
Week 2	148 ± 11	154 ± 4	158 ± 4	171 ± 5	ns
Week 4	140 ± 5	134 ± 7	161 ± 4	169 ± 10	+
Week 6	111 ± 5	97 ± 8	156 ± 11	187 ± 24	*,+++
Week 8	92 ± 8	117 ± 7	200 ± 7	153 ± 11	***
**Plasma NH**_**4**_^**+**^** (μM)**					
Week 0	61.2 ± 3.5	68.2 ± 8.5	68.9 ± 8.9		ns
Week 2	80.4 ± 5.5	93 ± 5.9	69.5 ± 11.3		ns
Week 4	106 ± 9.2	103.7 ± 10.9	50.9 ± 0.6		*
Week 6	126.4 ± 10.1	103.0 ± 8.1	60.6 ± 3.6		*
Week 8	152.9 ± 26.0	124.6 ± 17.1	53.9 ± 2.8		**,+
**Plasma bilirubin (mg/dl)**					
Week 0	< 0.5	< 0.5	< 0.5	< 0.5	ns
Week 2	4.7 ± 0.2	5.8 ± 0.5	< 0.5	< 0.5	ns
Week 4	6.2 ± 0.3	6.6 ± 0.3	< 0.5	< 0.5	ns
Week 6	6.9 ± 0.5	7.1 ± 0.4	< 0.5	< 0.5	ns

Plasma NH_4_^+^ showed no difference between shams and shams + probiotic. Therefore, we pooled them together for the analysis. P value: for plasma glucose and plasma NH_4_^+^, one-way ANOVA with Bonferroni correction, *significance between BDL and sham groups, ^+^significance between BDL + probiotic and sham groups. *ns* non-significant. For plasma bilirubin, one-way ANOVA with Bonferroni correction statistics are shown between BDL and BDL + probiotic.

### ^1^H MRS: BDL + probiotic rats display an attenuated rise in brain Gln

**Figure 2:**
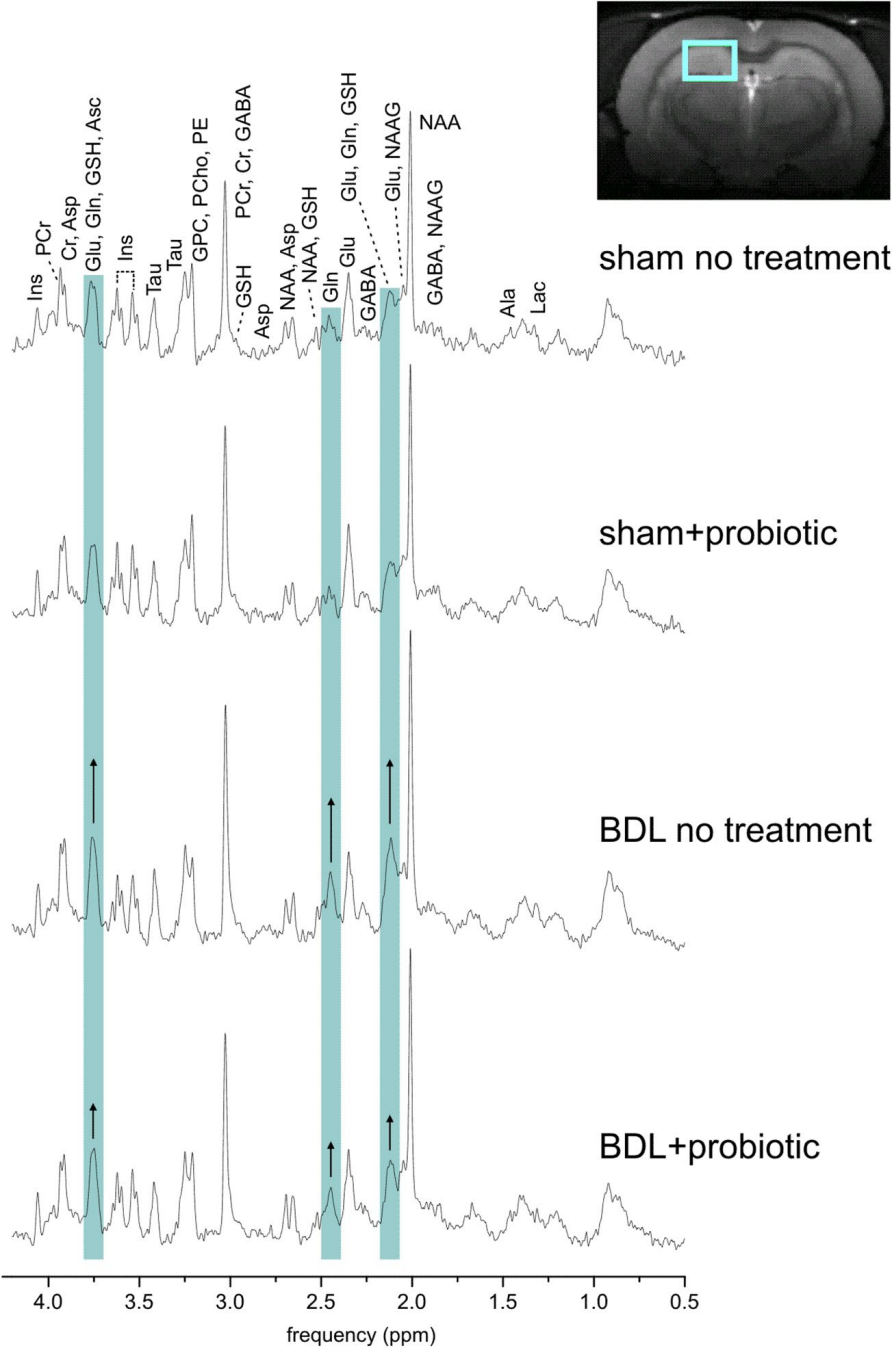
Representative in vivo ^1^H MRS spectra 8 weeks after surgery from sham rat, sham + probiotic rat, BDL rat and BDL + probiotic rat (SPECIAL sequence, TE = 2.8 ms, TR = 4000 ms, 160 averages). Blue bands highlight glutamine resonance that is visibly increased in BDL rats with smaller increase in BDL + probiotic rat. T_2_ weighted axial image of the rat brain indicates position of measured volume (2 × 2.8 × 2mm^3^) placed in dorsal hippocampus. *Ala *alanine, *Asc* ascorbate, *Asp* aspartate, *GPC* glycerophosphocholine, *PCho* phosphocholine, *Cr* creatine, *PCr* phosphocreatine, *GABA* γ-aminobutyric acid, *Glc* glucose, *Gln* glutamine, *Glu* glutamate, *GSH* glutathione, *mIns* myo-inositol, *Lac* lactate, *NAA N*-acetylaspartate, *NAAG N*-acetylaspartylglutamate, *PE* phosphoethanolamine, *Tau* taurine.

**Figure 3:**
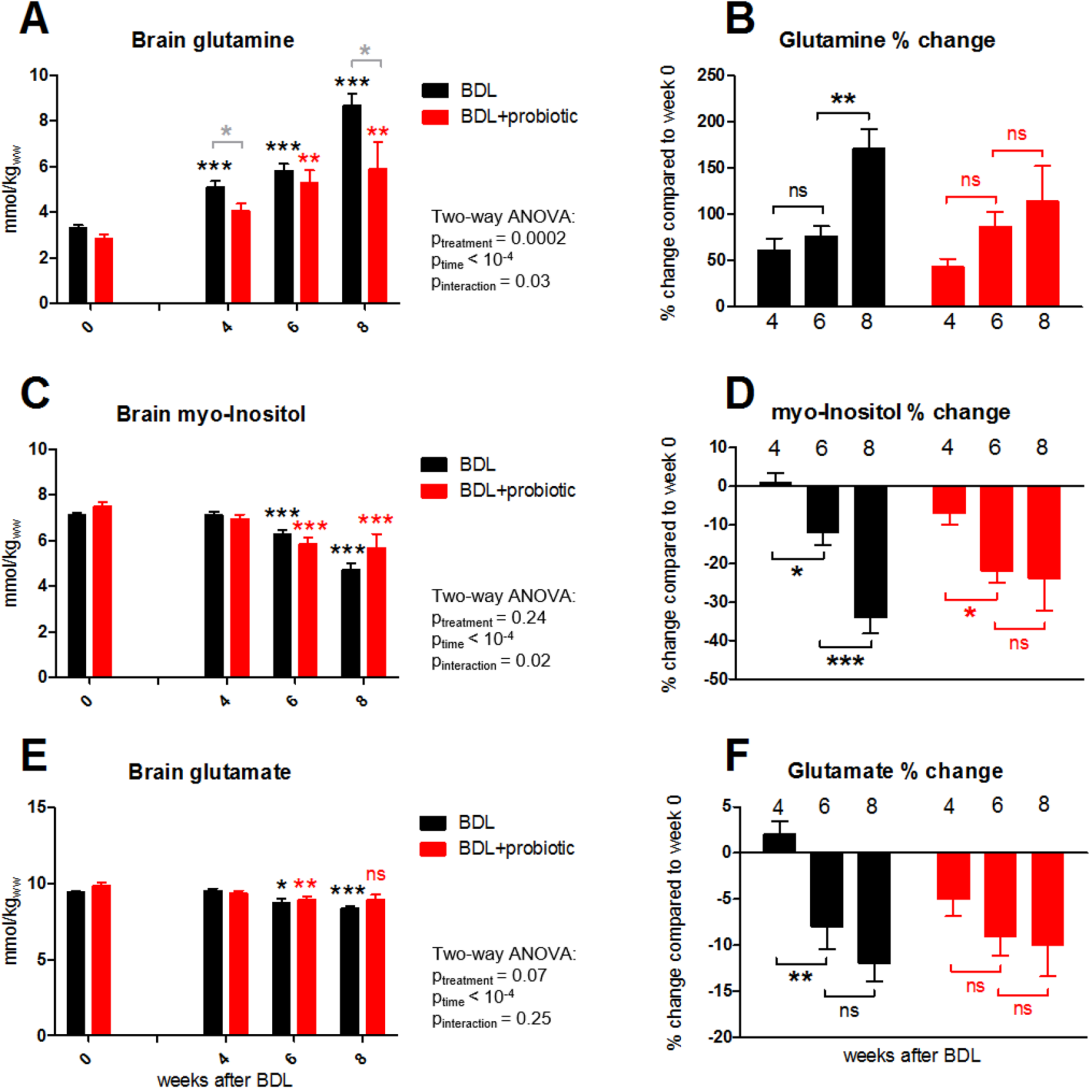
Longitudinal brain Gln, mIns and Glu. Expressed in absolute concentration as mmol/kg_ww_ (mmol per kg wet weight) (**A**,**C**,**E**) and % changes as compared to scan 0 (**B**,**D**,**F**). Significance (one-way ANOVA with Bonferroni corrections) on graphs (**A**,**C**,**E**) in comparison to brain concentration of corresponding metabolite at week 0 (black, red) or between the two groups for a given week (grey). *ns* non-significant.

The spectral quality with visible increase in brain Gln in BDL rats is shown on Fig. 2. Brain **Gln** increased in both BDL and BDL + probiotic rats, but the BDL + probiotic group displayed a significantly slower and attenuated increase (Fig. 3A,B). BDL rats showed a significant + 61 ± 13% increase of Gln already 4 weeks post-BDL, reaching + 171 ± 22% at week 8. In BDL + probiotic the rise in Gln occurred later, becoming significant only at 6 weeks post-BDL and reaching only + 114 ± 38% increase at week 8.

Gln increase was followed by **mIns** decrease in both BDL and BDL + probiotic groups. In BDL rats, mIns decreased significantly by − 34 ± 4% at 8 weeks post-BDL (Fig. 3C,D). The decrease in BDL + probiotic was less pronounced (− 24 ± 8%), which was expected since Gln increase was lesser in BDL + probiotic, given that a mIns decrease is generally considered as an osmoregulatory response to Gln increase.

Among the other brain organic osmolytes, **tCho** and **Tau** decreased in both BDL and BDL + probiotic, and there was no significant difference in their decrease between BDL and BDL + probiotic group at 8 weeks post-BDL (Table 3). tCho decreased by − 13 ± 11% at 8 weeks post-BDL in BDL group and by − 22 ± 8% in BDL + probiotic. Tau decreased by − 7 ± 2% in BDL group and − 13 ± 2% in BDL + probiotic at 8 weeks post-BDL.

**Table 3:** Concentration changes of some brain metabolites 8 weeks after BDL surgery in BDL and BDL + probiotic groups.

Brain metabolite	Concentration changes 8 weeks after BDL						BDL vs BDL + probiotic
	BDL			BDL + probiotic			
	Mean (%)	SEM (%)	p value^1^	mean (%)	SEM (%)	p value^1^	p value^2^
tCho	− 13	11	ns	− 22	8	0.04	ns
Tau	− 7	2	0.005	− 13	2	0.0004	ns
Cr	− 7	3	0.05	− 12	4	0.05	ns
PCr	− 12	4	0.01	− 15	3	0.002	ns
tCr	− 10	1	0.0001	− 14	3	0.002	ns
Asc	− 12	8	ns	− 18	8	0.05	ns

^1^One-way ANOVA repeated measure between concentrations of corresponding metabolite at week 0 and week 8 after BDL surgery.

^2^One-way ANOVA between % changes of corresponding metabolite in BDL and BDL + probiotic groups.

The neurotransmitter **Glu** decreased in both BDL and BDL + probiotic groups, but the decrease was slightly less pronounced in BDL + probiotic rats (− 10 ± 3.4% in BDL + probiotic vs − 12 ± 1.9% in BDL rats at 8 weeks post-BDL) (Fig. 3E,F). The decrease in other brain metabolites (**Cr, PCr, tCr, Asc**) was not significantly different between BDL and BDL + probiotic groups and their evolution at 8 weeks post-BDL is shown in Table 3. In addition, no significant changes were observed in **Ala, Asp, GABA, GSH, PE, NAA, NAAG nor tNAA** during the progression of disease neither in BDL or BDL + probiotic group. Finally, there was no significant differences observed between shams and sham + probiotic group for any given metabolite (data not shown).

### Behavioural tests: better performance in BDL + probiotic rats

**Figure 4:**
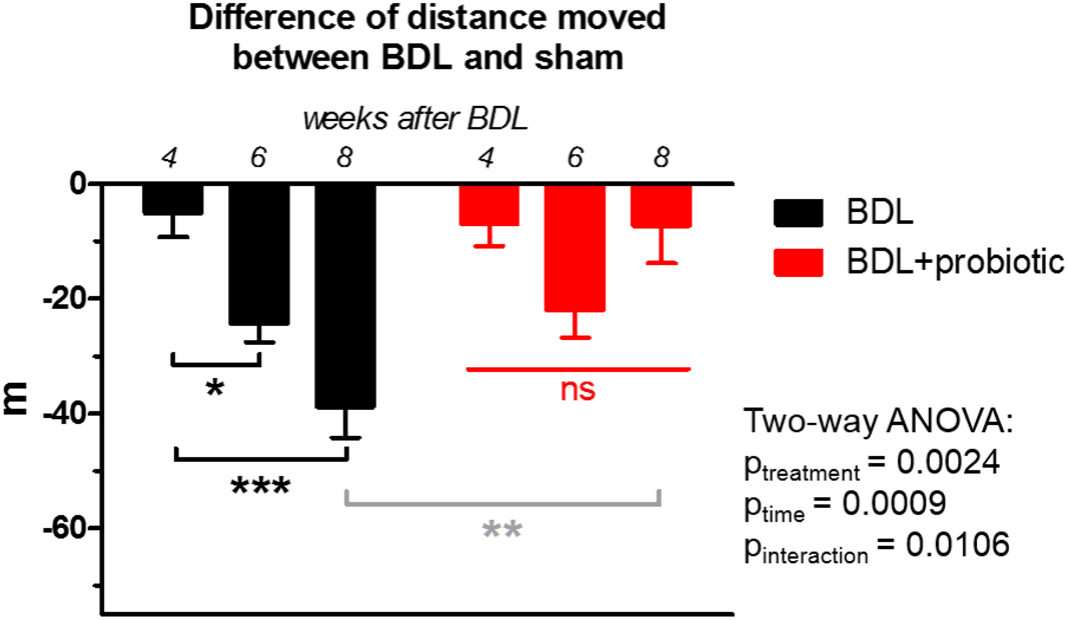
Performance in open field test. In black, difference in distance moved in meters (m) during the open field test between BDL rats and their shams at corresponding weeks. In red, difference between BDL + probiotic rats and their shams (sham + probiotic). There was no significant change between any weeks in BDL + probiotic group. Significance (one-way ANOVA with Bonferroni corrections) is given intra-group (black, red) or between the two groups at week 8 (grey).

BDL + probiotic group also exhibited a better performance in the Open Field test compared to BDL group (Fig. 4). Eight weeks post-BDL, the BDL group walked 38.8 ± 5.3 m less that their shams during the 10 min Open Field test. On the other hand, BDL + probiotic rats displayed stable performance between weeks 4, 6 and 8 post-BDL compared to shams, walking only 7.3 ± 6.5 m less that the shams at week 8. This difference at week 8 was significant between BDL and BDL + probiotic group. There were no differences for the following: time spent in the wall-zone, centre-zone and inter-zone, and latency to enter centre zone, suggesting animal anxiety was not a variable (data not shown).

## Discussion

In the present study, we showed that prolonged administration of a multistrain probiotics mixture (VIVOMIXX) in a rat model of type C HE resulted in a milder course of HE compared to untreated animals. The administration of probiotics reduced plasma ammonium in BDL rats and was associated with milder changes in the neurometabolic profile compared to BDL rats without probiotics. Both the neurometabolic profile of BDL + probiotic rats and their performance in behavioral tests were improved at week 8 post-BDL compared to the BDL group without treatment.

A lower plasma ammonium concentration was observed in BDL + probiotic group, possibly related to the modification of gut flora by the probiotic bacteria. The probiotic mixture used in our study led to an increase in *Bifidobacteria* in the gut of treated rats, both in shams and BDL, as shown in Fig. 1. This probably reflects the ability of probiotics to alter the microbiota composition in the gut. In BDL rats, such an alteration can decrease urea-derived production and absorption of ammonium, in agreement with previous studies where decreased ammonium has been linked to probiotics utilization^29–31^. Furthermore, probiotics may also reduce plasma ammonium by decreasing intestinal permeability, improving the gut epithelial integrity or increasing the ability of the liver to detoxify ammonium^32^. It has been shown that the autochthonous taxa of gut bacteria can reduce endotoxemia, intestinal inflammation, and nourish colonocytes by producing short-chain fatty acids and maintain good intestinal barrier^32,51^. Moreover, probiotic supplementation was also shown to decrease systemic inflammation^52^ and production of gut-derived bacterial toxins^28^, and to reduce risk of hospitalization of cirrhotic patients^53^.

As previously mentioned, the efficacy of probiotics on HE has been mainly assessed through neurological testing. Some studies have shown that probiotics are effective in improving minimal symptoms or progression to overt HE as compared to placebo^27,36,54,55^. In the present study, a significantly better performance in behavioural tests was observed in BDL + probiotic group compared to the BDL group without treatment, which coincided with attenuated neurometabolic changes compared to untreated animals. In particular, the brain Gln was lesser with a consequently milder mIns response.

Gln is probably the first metabolite influenced by increased plasma ammonium^43^, and both are considered responsible for many of the changes in HE. Also, it is commonly accepted in chronic HE that increased plasma ammonium generates a rise in the brain osmolyte Gln leading to osmotic imbalance followed secondarily by a partial compensation through the gradual decrease of other brain osmolytes^43,56^. The observed effect of probiotic treatment in our study could be attributed to lower plasma levels of ammonium with consequently lower brain Gln and mIns concentrations. Of course, the effect on both gut- and systemic inflammation may also have contributed, something warranting further studies.

We also measured a smaller decrease in the neurotransmitter Glu in the BDL + probiotic group compared to the untreated BDL rats. It is now well known that HE is linked to disturbances in the neurotransmission systems (including glutamatergic among others) although their direct role is still not fully understood^57^. This smaller Glu decrease may also have been secondary to the attenuated Gln increase in the treated rats, given that Gln synthesis is connected with Glu through the glutamate-glutamine cycle. Moreover, our previous studies have shown that the reduction in the cytosolic pool of Glu in BDL rats could simply be the result of ammonium detoxification driven by increased Gln synthesis from Glu in astrocytes without consequences on neurotransmission^58^.

The present study has some limitations. We focused on the assessment of brain metabolites changes in BDL rats receiving VIVOMIXX. As such, no experiments assessing systemic or central inflammation were performed, something which would need further investigation in future studies. In addition, the increase in *Bifidobacteria* in the gut of both shams and BDL-treated rats observed in our study needs further investigation since the number of samples for gut microbiota measurements were limited.

In conclusion, the administration of a specific probiotic formulation (VIVOMIXX) in a prophylactic manner (treatment started before the beginning of the disease) had a beneficial effect on the development of HE in a rat model of type C HE, both when analysing locomotor activity and neurometabolic profile. It reduced or delayed disease progression, probably by decreasing plasma ammonium which may be related to increasing *Bifidobacteria* in the gut and slowing down the occurrence of precipitating factors. These are positive findings as some of these metabolic changes in the brain reflect disease severity^41^. Recently, it was shown that MRS changes (Glx, Ins, tCho) in cirrhotic patients were correlated to changes in gut microbiota^11^. Taken together, these results are promising and warrant further investigation. The clinical implications of these findings are potentially far-reaching given that probiotics are generally safe and well-tolerated by patients^26,59,60^ in contrast to lactulose^61,62^.

## References

[1] Dasarathy S (2017). Ammonia toxicity: From head to toe?. Metab. Brain Dis..

[2] Romero-Gómez M, Jover M, Galán JJ, Ruiz A (2009). Gut ammonia production and its modulation. Metab. Brain Dis..

[3] Weiss N, Jalan R, Thabut D (2018). Understanding hepatic encephalopathy. Intensive Care Med..

[4] Collins CM, D’Orazio SEF (1993). Bacterial ureases: Structure, regulation of expression and role in pathogenesis. Mol. Microbiol..

[5] Bajaj JS (2014). The role of microbiota in hepatic encephalopathy. Gut Microbes.

[6] Hansen BA, Vilstrup H (1985). Increased intestinal hydrolysis of urea in patients with alcoholic cirrhosis. Scand. J. Gastroenterol..

[7] Chen Y (2011). Characterization of fecal microbial communities in patients with liver cirrhosis. Hepatology.

[8] Bajaj JS (2012). Linkage of gut microbiome with cognition in hepatic encephalopathy. AJP Gastrointest. Liver Physiol..

[9] Bajaj JS (2014). Altered profile of human gut microbiome is associated with cirrhosis and its complications. J. Hepatol..

[10] Sabino J (2016). Primary sclerosing cholangitis is characterised by intestinal dysbiosis independent from IBD. Gut.

[11] Ahluwalia V (2016). Impaired gut-liver-brain axis in patients with cirrhosis. Sci. Rep..

[12] Kakiyama G (2013). Modulation of the fecal bile acid profile by gut microbiota in cirrhosis. J. Hepatol..

[13] Delahooke DM, Barclay GR, Poxton IR (1995). A Reappraisal of the biological-activity of bacteroides Lps. J. Med. Microbiol..

[14] Bajaj JS (2012). Colonic mucosal microbiome differs from stool microbiome in cirrhosis and hepatic encephalopathy and is linked to cognition and inflammation. AJP Gastrointest. Liver Physiol..

[15] Llorente C, Schnabl B (2015). The gut microbiota and liver disease. Cell. Mol. Gastroenterol. Hepatol..

[16] Garcia-Tsao G (1995). Bacterial translocation to mesenteric lymph-nodes is increased in cirrhotic rats with ascites. Gastroenterology.

[17] Saitoh O (1999). Increased prevalence of intestinal inflammation in patients with liver cirrhosis. World J. Gastroenterol..

[18] Du Plessis J (2013). Activated intestinal macrophages in patients with cirrhosis release NO and IL-6 that may disrupt intestinal barrier function. J. Hepatol..

[19] Wiest R, Lawson M, Geuking M (2014). Pathological bacterial translocation in liver cirrhosis. J. Hepatol..

[20] Wiest R, Lawson M, Geuking M (2014). Reply to: ‘Bacterial translocation in liver cirrhosis: Site and role in fibrogenesis’. J. Hepatol..

[21] Patel VC, White H, Støy S, Bajaj JS, Shawcross DL (2016). Clinical science workshop: Targeting the gut-liver-brain axis. Metab. Brain Dis..

[22] Merli M (2010). Cirrhotic patients are at risk for health care-associated bacterial infections. Clin. Gastroenterol. Hepatol..

[23] Quigley EMM, Stanton C, Murphy EF (2013). The gut microbiota and the liver. Pathophysiological and clinical implications. J. Hepatol..

[24] Clausen MR, Mortensen PB (1997). Lactulose, disaccharides and colonic flora. Clinical consequences. Drugs.

[25] Bajaj JS (2013). Modulation of the metabiome by rifaximin in patients with cirrhosis and minimal hepatic encephalopathy. PLoS ONE.

[26] Ziada DH, Soliman HH, El Yamany SA, Hamisa MF, Hasan AM (2013). Can Lactobacillus acidophilus improve minimal hepatic encephalopathy? A neurometabolite study using magnetic resonance spectroscopy. Arab. J. Gastroenterol..

[27] Shukla S, Shukla A, Mehboob S, Guha S (2011). Meta-analysis: The effects of gut flora modulation using prebiotics, probiotics and synbiotics on minimal hepatic encephalopathy. Aliment. Pharmacol. Ther..

[28] Rowland I (2010). Current level of consensus on probiotic science-report of an expert meeting—London, 23 November 2009. Gut Microbes.

[29] Pratap Mouli V (2014). Effect of probiotic VSL#3 in the treatment of minimal hepatic encephalopathy: A non-inferiority randomized controlled trial. Hepatol. Res..

[30] Sharma P, Sharma BC, Puri V, Sarin SK (2008). An open-label randomized controlled trial of lactulose and probiotics in the treatment of minimal hepatic encephalopathy. Eur. J. Gastroenterol. Hepatol..

[31] Pereg D (2011). Probiotics for patients with compensated liver cirrhosis: A double-blind placebo-controlled study. Nutrition.

[32] Nava GM, Stappenbeck TS (2011). Diversity of the autochthonous colonic microbiota. Gut Microbes.

[33] Mittal VV, Sharma BC, Sharma P, Sarin SK (2011). A randomized controlled trial comparing lactulose, probiotics, and l-ornithine l-aspartate in treatment of minimal hepatic encephalopathy. Eur. J. Gastroenterol. Hepatol..

[34] Lunia MK, Sharma BC, Sharma P, Sachdeva S, Srivastava S (2014). Probiotics prevent hepatic encephalopathy in patients with cirrhosis: A randomized controlled trial. Clin. Gastroenterol. Hepatol..

[35] Román E (2019). Effect of a multistrain probiotic on cognitive function and risk of falls in patients with cirrhosis: A randomized trial. Hepatol. Commun..

[36] Saab S (2016). Probiotics are helpful in hepatic encephalopathy: A meta-analysis of randomized trials. Liver Int..

[37] Ding X, Zhang F, Wang Y (2014). Letter: Probiotics vs lactulose for minimal hepatic encephalopathy therapy. Aliment. Pharmacol. Ther..

[38] Pratap Mouli V (2015). Effect of probiotic VSL#3 in the treatment of minimal hepatic encephalopathy: A non-inferiority randomized controlled trial. Hepatol. Res..

[39] Chavarria L, Cordoba J (2013). Magnetic resonance of the brain in chronic and acute liver failure. Metab. Brain Dis..

[40] Keiding S, Pavese N (2013). Brain metabolism in patients with hepatic encephalopathy studied by PET and MR. Arch. Biochem. Biophys..

[41] Grover V-PB (2006). Current and future applications of magnetic resonance imaging and spectroscopy of the brain in hepatic encephalopathy. World J. Gastroenterol..

[42] Rackayova V (2016). 1H and 31P magnetic resonance spectroscopy in a rat model of chronic hepatic encephalopathy: In vivo longitudinal measurements of brain energy metabolism. Metab. Brain Dis..

[43] Braissant O (2019). Longitudinal neurometabolic changes in the hippocampus of a rat model of chronic hepatic encephalopathy. J. Hepatol..

[44] D’Mello C (2015). Probiotics improve inflammation-associated sickness behavior by altering communication between the peripheral immune system and the brain. J. Neurosci..

[45] Hammes TO (2017). Lactobacillus rhamnosusGG reduces hepatic fibrosis in a model of chronic liver disease in rats. Nutr. Hosp..

[46] Dalal R, McGee RG, Riordan SM, Webster AC (2017). Probiotics for people with hepatic encephalopathy. Cochrane Database Syst. Rev.

[47] Butterworth RF (2009). Experimental models of hepatic encephalopathy: ISHEN guidelines. Liver Int..

[48] Mastromarino P (2014). Correlation between lactoferrin and beneficial microbiota in breast milk and infant’s feces. Biometals.

[49] Mlynárik V, Gambarota G, Frenkel H, Gruetter R (2006). Localized short-echo-time proton MR spectroscopy with full signal-intensity acquisition. Magn. Reson. Med..

[50] Bahceci F, Yildirim B, Karincaoglu M, Dogan I, Sipahi B (2005). Memory impairment in patients with cirrhosis. J. Natl. Med. Assoc..

[51] Dabard J (2001). Ruminococcin A, a new lantibiotic produced by a *Ruminococcus gnavu* s strain isolated from human feces. Appl. Environ. Microbiol..

[52] Stadlbauer V (2008). Effect of probiotic treatment on deranged neutrophil function and cytokine responses in patients with compensated alcoholic cirrhosis. J. Hepatol..

[53] Dhiman RK (2014). Probiotic VSL#3 reduces liver disease severity and hospitalization in patients with cirrhosis: A randomized, controlled trial. Gastroenterology.

[54] Ding X, Zhang F, Wang Y (2014). Letter: Probiotics vs lactulose for minimal hepatic encephalopathy therapy. Alimentary Pharmacol. Therap..

[55] McGee RG, Bakens A, Wiley K, Riordan SM, Webster AC (2011). Probiotics for patients with hepatic encephalopathy. Cochrane Database Syst. Rev..

[56] Córdoba J (2001). The development of low-grade cerebral edema in cirrhosis is supported by the evolution of (1)H-magnetic resonance abnormalities after liver transplantation. J. Hepatol..

[57] Felipo V (2013). Hepatic encephalopathy: Effects of liver failure on brain function. Nat. Rev. Neurosci..

[58] Baker L (2016). New technologies—new insights into the pathogenesis of hepatic encephalopathy. Metab. Brain Dis..

[59] Holte K, Krag A, Gluud LL (2012). Systematic review and meta-analysis of randomized trials on probiotics for hepatic encephalopathy. Hepatol. Res..

[60] Bajaj JS (2014). Randomized clinical trial: Lactobacillus gg modulates gut microbiome, metabolome and endotoxemia in patients with cirrhosis. Aliment Pharmacol. Ther..

[61] Sharma BC, Singh J (2016). Probiotics in management of hepatic encephalopathy. Metab. Brain Dis..

[62] Patidar KR, Bajaj JS (2015). Covert and overt hepatic encephalopathy: Diagnosis and management. Clin. Gastroenterol. Hepatol..

